# Delayed hydronephrosis due to retroperitoneal hematoma after a seatbelt injury: A case report

**DOI:** 10.1097/MD.0000000000011022

**Published:** 2018-06-18

**Authors:** Tetsuya Yumoto, Yoshitaka Kondo, Kento Kumon, Yoshihisa Masaoka, Takao Hiraki, Taihei Yamada, Hiromichi Naito, Atsunori Nakao

**Affiliations:** a Advanced Emergency and Critical Care Medical Center, Okayama University Hospital; b Department of Gastroenterological Surgery, Okayama University Graduate School of Medicine, Dentistry and Pharmaceutical Sciences; c Department of Radiology, Okayama University Medical School, Okayama, Japan.

**Keywords:** blunt abdominal trauma, hydronephrosis, retroperitoneal hematoma, seatbelt injury

## Abstract

**Rationale:**

Hydronephrosis caused by retroperitoneal hematoma after a seatbelt injury is a unique clinical entity.

**Patient concerns:**

A 21-year-old man, who had been wearing a seatbelt, was brought to our hospital after a motor vehicle collision, complaining of abdominal pain. Computed tomography (CT) revealed retroperitoneal hematoma in the upper pelvic region. Since he was hemodynamically stable throughout admission, he was managed conservatively. Seventeen days after initial discharge, the patient revisited our emergency department due to right back pain.

**Diagnoses:**

CT scans indicated retroperitoneal hematoma growth resulting in hydronephrosis of the right kidney.

**Interventions:**

Laparoscopic drainage of the retroperitoneal hematoma was successfully performed.

**Outcomes:**

His symptoms resolved after the surgery. Follow-up CT scans three months later demonstrated complete resolution of the hydronephrosis and retroperitoneal hematoma.

**Lessons:**

Our case highlights a patient with delayed hydronephrosis because of retroperitoneal hematoma expansion after a seatbelt injury.

## 1 Introduction

The effectiveness of wearing a seat belt on reducing traffic fatalities has been well established^[1]^; however, various types of abdominal injuries have been caused by seatbelt use, including solid organ injuries, bowel perforation, mesenteric avulsion, abdominal wall disruption, abdominal aortic rupture, chance fractures of the spine, and combinations of these.^[2–4]^

Retroperitoneal hemorrhage unrelated to pelvic fracture following blunt abdominal trauma can occur after injuries to major vessels or solid or hollow organs contained in the retroperitoneal space.^[5]^ Although blunt injury to the major abdominal vessels, including abdominal aortic injury, is extremely rare,^[6]^ retroperitoneal hematoma after seatbelt injury progressing into ureter compression accompanied by hydronephrosis has never been reported. Here, we describe a unique case of retroperitoneal hemorrhage associated with a seatbelt-related injury, which caused subsequent hydronephrosis successfully treated with laparoscopic surgery.

## 2 Methods

Written informed consent was obtained from the patient for publication of this case report. The ethical approval was not required in our hospital because it was a case report without any research involving human beings or experimental subjects.

## 3 Case presentation

**Figure 1:**
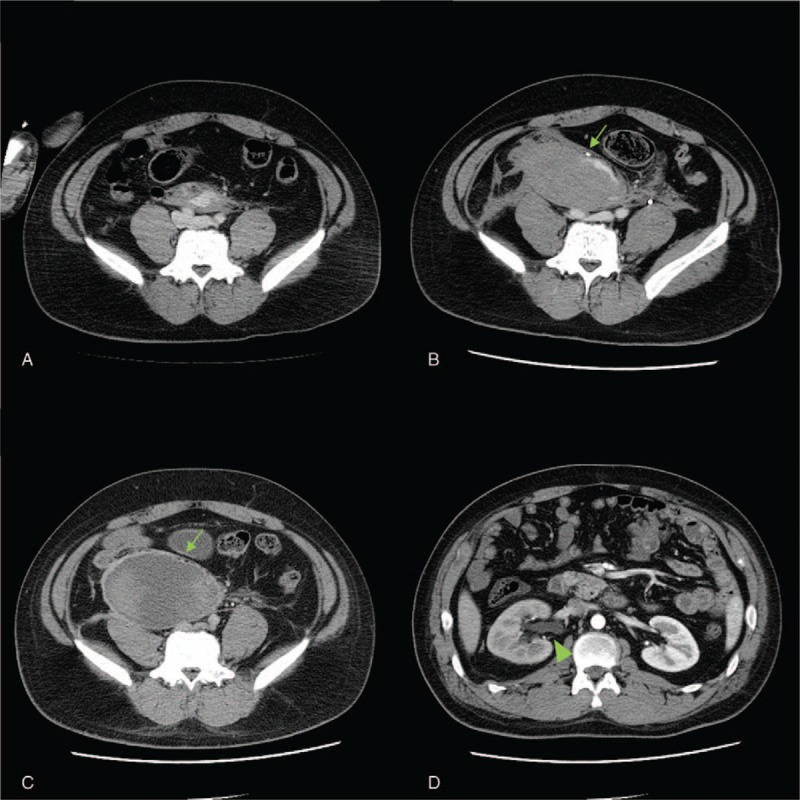
Time course of retroperitoneal hematoma on contrast-enhanced CT in the portal venous phase. Contrast extravasation was visible in front of the aortic bifurcation (A: on arrival). Contrast extravasation and hematoma expansion lifting the right ureter (arrow) was identified (B: 6 hours after arrival). The compressed right ureter (arrow) and dilatation of renal pelvis (triangle arrow) caused by hematoma growth was identified. (C, D: 24 days after injury). CT = computed tomography.

**Figure 2:**
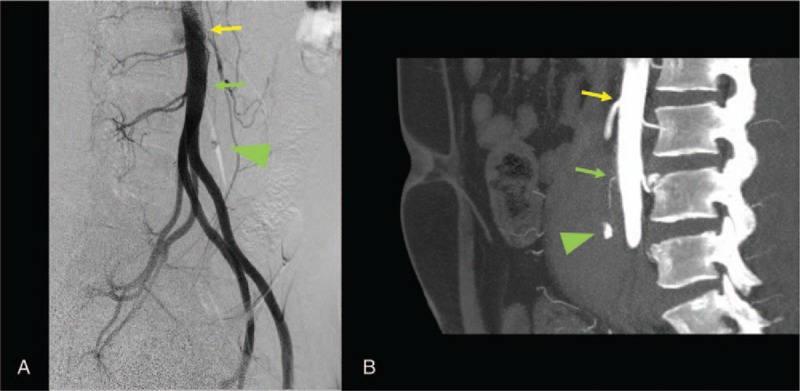
Right oblique image of aortography (A) and left lateral image of CT aortography (B) 6 hours after injury showing contrast extravasation (triangle arrow). The affected vessel arose from the anterior wall of the abdominal aorta (yellow arrow) 41 mm below the origin of the inferior mesenteric artery (green arrow). CT = computed tomography.

A 21-year-old man was transferred to our emergency department after a head-on motor vehicle collision. He was wearing a 3-point seatbelt and his car's front airbag had deployed. The patient had no significant medical history, but was later diagnosed with neurofibromatosis 1 (NF1) based on the finding of multiple café au lait macules and subcutaneous tumors on his back. On arrival at the emergency department, he complained of lower abdominal pain. An impression of the seat belt was evident across his lower abdomen, where he had moderate tenderness without guarding or rebound tenderness. His vital signs were as follows: Glasgow Coma Scale score, 14 (E4V4M6); respiratory rate, 18 breaths/min; pulse rate, 96 beats/min; and blood pressure, 148/100 mmHg. Focused assessment with sonography for trauma excluded any intra-abdominal effusion. Contrast-enhanced computed tomography (CT) revealed retroperitoneal hematoma with contrast extravasation above the level of the bifurcation of the abdominal aorta (Fig. 1A). Neither intra-abdominal organ injury, fluid collection, nor pelvic fractures were observed. Since he was hemodynamically stable, he was admitted to the emergency intensive care unit for close observation. Follow-up contrast-enhanced CT scans performed 6 hours after the initial scans identified enlargement of the retroperitoneal hematoma with contrast extravasation (Fig. 1B). Although the patient remained hemodynamically stable, we decided to move him to the angiography suite to stop the bleeding. An aortography detected an affected vessel originating from the anterior wall of the abdominal aorta 41 mm below the origin of the inferior mesenteric artery (Fig. 2A and B). However, it was difficult to catheterize the affected vessel. Since the patient had remained hemodynamically stable without receiving blood transfusions, we expected spontaneous resolution of the retroperitoneal hemorrhage. Hematoma size expansion was not observed on CT scans the next day. The patient was discharged from the hospital on day 7 when his abdominal pain had almost subsided. Follow-up CT was scheduled 1 month after discharge.

**Figure 3:**
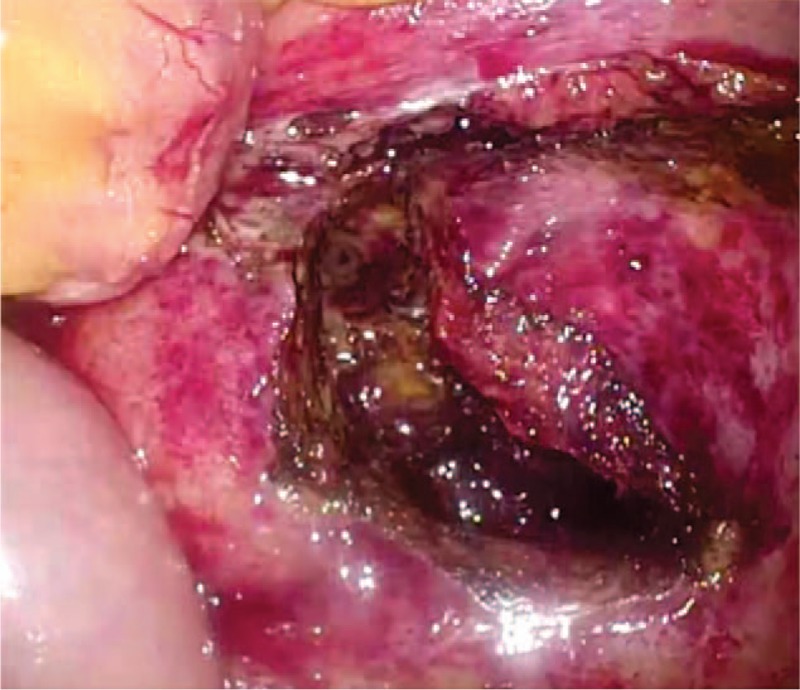
Intraoperative photography from the laparoscopic drainage of retroperitoneal hematoma. A thickened retroperitoneum filled with fibrinous clot without active bleeding was observed.

He presented to our emergency department again 17 days after discharge due to progressive back pain on the right side for a few days. He had costovertebral angle tenderness on his right side. Laboratory data revealed an elevated serum creatinine concentration of 1.03 mg/dL from 0.69 mg/dL. Contrast-enhanced CT scans indicated the retroperitoneal hematoma growth had resulted in hydronephrosis caused by ureteral compression on the right side (Fig. 1C and D). As his symptoms and vital signs were stable, laparoscopic drainage of the retroperitoneal hematoma was performed 4 days after readmission. Laparoscopic surgery revealed a retroperitoneal hematoma extending from the dorsal area of the cecum to the pelvis. More than 400 mL of old hematoma was drained through the small retroperitoneal incision. Subsequently, the incision was extended to explore inside the cavity. Laparoscopy detected an oozing point on the inner surface of the cavity, and the bleeding vessel presumed to be responsible was successfully ablated (Fig. 3). Hemostatic agent was packed in the cavity after ablation of the oozing point. The incision of the retroperitoneum was closed by running suture. Although the patient developed postoperative ileus, he was discharged 1 month after the surgery. Follow-up CT scans 3 months later demonstrated complete resolution of the hydronephrosis and retroperitoneal hematoma.

## 4 Discussion

This patient's course provides 2 important clinical implications. First, seatbelt injury can cause an unusual retroperitoneal hemorrhage resulting in delayed presentation of hydronephrosis secondary to ureteral compression. Second, retroperitoneal hematoma can be treated successfully with laparoscopic drainage.

Retroperitoneal hemorrhage was detected in 12% of hemodynamically stable blunt abdominal trauma patients receiving CT scans.^[7,8]^ Retroperitoneal visceral organs or major vascular injuries are responsible for retroperitoneal hemorrhage unrelated to pelvic fractures, which can be potentially overlooked since focused assessment of sonography for trauma fails to detect retroperitoneal hematoma.^[9,10]^ The abdominal aorta, inferior vena cava, iliac artery or vein, superior mesenteric artery, and portal vein have been reported in common intraabdominal vascular injuries in patients with penetrating trauma.^[11]^ However, little data are available regarding blunt abdominal vascular injuries. Zone 3, which represents the region from the lowest renal artery to the aortic bifurcation, was the most common site of blunt abdominal aortic injuries, which range from minimum aortic injury to aortic rupture.^[12]^ Hemodynamically unstable patients with retroperitoneal hemorrhage warrant immediate intervention. Angioembolization is effective in stopping bleeding in zone 3 retroperitoneal hematoma; otherwise, exploratory laparotomy can cause fatal uncontrollable hemorrhage.^[13]^ In the present case, artery embolization was attempted after identification of ongoing bleeding signs on follow-up CT scans, which resulted in unsuccessful catheterization of the affected vessel. Nonoperative management was eventually selected because the patient was hemodynamically stable without obvious findings of injuries to any other organs.

The mechanism of blunt abdominal aortic injury in our case was thought to be sudden deceleration force or direct seatbelt compression to the vertebrae.^[12]^ Vascular abnormalities, including stenosis, aneurysms, or even spontaneous arterial rupture, have been described in patients with NF1.^[14,15]^ Arterial dysplasia is considered to cause vascular fragility.^[14,15]^ In the present case, vascular fragility due to the presence of NF1 may also have contributed to the isolated arterial injury, resulting in the unusual pattern of retroperitoneal hemorrhage. Chronic expanding hematoma, which mostly results from trauma or bleeding disorder, has been sporadically reported; only 1 case of retroperitoneal hematoma without a history of trauma presenting with hydronephrosis has been described.^[16]^

Trauma laparoscopy is useful in identifying retroperitoneal hematoma.^[17]^ Either the transperitoneal or retroperitoneal route has been reported as a safe approach in patients with renal cell carcinoma who had undergone partial nephrectomy.^[18]^ In the present case, the transperitoneal laparoscopic approach was selected, considering the risk of disorientation, which may cause inadvertent injury.^[19]^ Furthermore, laparoscopy was useful for intraoperative identification of the affected small vessel that originated directly from the abdominal aorta, which was detected with preoperative CT imaging.

To the best of our knowledge, this is the first report of an unusual pattern of retroperitoneal hematoma after seatbelt injury leading to delayed presentation of hydronephrosis. If the affected artery had been successfully embolized initially, subsequent hydronephrosis might not have occurred. Nevertheless, even if a patient is hemodynamically stable and managed conservatively as in our case, careful attention should be paid to whether hematoma expansion occurs.

## 5 Conclusions

We report a unique case of retroperitoneal hematoma following seatbelt injury, which subsequently evolved into hydronephrosis due to ureteral compression. Careful follow-up is required to evaluate the presence of hematoma expansion that may potentially cause hydronephrosis. Laparoscopic drainage of retroperitoneal hematoma, if needed, may be a safe and effective procedure.

## Author contributions

**Conceptualization:** Tetsuya Yumoto.

**Supervision:** Atsunori Nakao.

**Writing – original draft:** Tetsuya Yumoto, Yoshitaka Kondo, Takao Hiraki.

**Writing – review and editing:** Kento Kumon, Yoshihisa Masaoka, Taihei Yamada, Hiromichi Naito, Atsunori Nakao.
